# Microstructure Evolution and Strengthening Mechanism of V-N Microalloyed Invar Alloy Processed by Mechanical Heat Treatment

**DOI:** 10.3390/ma18173934

**Published:** 2025-08-22

**Authors:** Shuo Zhang, Xueting Liu, Hui Liu, Yanchong Yu, Cong Chang, Yanjun Di

**Affiliations:** 1 College of Materials Science and Engineering, Taiyuan University of Technology, Taiyuan 030024, China; zhangshuo0661@link.tyut.edu.cn (S.Z.); liuxueting202302@163.com (X.L.); liuh0706@163.com (H.L.); 2 Gansu Jiugang Steel Group Hongxing Iron and Steel Co., Ltd., Jiayuguan 735100, China; diyanjun@jiugang.com

**Keywords:** invar alloy, V (C, N), deformation strengthening, comprehensive strengthening mechanism, low thermal expansion properties

## Abstract

Invar alloy is widely used for manufacturing precision instruments owing to its exceptionally low thermal expansion property. Nevertheless, conventional invar alloys usually lack the sufficient strength required for engineering applications, so there is an urgent need for innovative approaches to enhance the strength. In this study, the possibility of elevating the strength and preserving the low coefficient of thermal expansion (CTE) of invar alloy is investigated by combining deformation strengthening with the V (C, N) precipitation strengthening mechanism. The increase in dislocation density resulting from deformation treatment promotes the V (C, N) precipitation. This leads to a significant enhancement in the strength of alloys after cold-rolling aging compared to direct aged counterparts, while preserving the low CTE. After a cold-rolling deformation with a 40% reduction and subsequent aging at 650 °C for 3 h, the V-N invar alloy exhibits a tensile strength of 907 MPa and an elongation of 6.9%, demonstrating the optimal mechanical properties. In addition, the CTE value maintains a low value of 1.31 × 10^−6^/°C within the temperature range of 20 to 100 °C. These findings are vital for developing high-strength, low-CTE invar alloys.

## 1. Introduction

Fe-36Ni invar alloys are widely utilized in precision instruments, large telescope bases, and metal masks due to their exceptionally low thermal expansion properties [1,2,3]. However, their inherently low strength limits their broader application in structurally and functionally integrated system. In recent years, with the expansion of application industries, such as long-distance multiple capacity wires and aerospace composite molds, there has been an increasing demand for invar alloys with higher mechanical properties [4,5]. Therefore, strengthening invar alloys while maintaining low thermal expansion characteristics is the key research focus.

Invar alloys produced using an emerging additive manufacturing process retain excellent thermal expansion characteristics, but typically suffer from low mechanical strength [6,7]. At present, the primary strengthening methods for invar alloys include solid solution strengthening [8], grain refinement [9,10], deformation strengthening [11,12], and precipitation strengthening [13,14]. Among these, carbide precipitation strengthening offers significant advantages in enhancing strength while simultaneously controlling the coefficient of thermal expansion (CTE). Nakama et al. [15] investigated the effects of various MC-type carbides (MC, where M represents a metal element such as V or Ti) on the aging hardness and CTE of Fe-36% Ni-0.2% C invar alloys. The results indicated that VC is particularly suitable for making high-strength invar alloys with a lower CTE. The precipitation strengthening effect is influenced by the size and number density of precipitated phases. This effect becomes more pronounced as the precipitates’ size decreases and number density increases [16,17]. Compared to single carbides, mixed precipitated phases exhibit finer sizes and slower coarsening rates due to the varying diffusion rates of constituent elements [18]. Promising progress has been made in strengthening invar alloys through composite precipitated phases, such as VC, Mo_2_C and TiN [11], Ti (C, N) and VC [19], and Mo_2_C and (V, Mo) C [20]. Numerous studies have demonstrated that nitrogen facilitates the formation of V (C, N) precipitates, which significantly enhance the alloy’s strength [21,22]. Furthermore, the high solubility of V (C, N) facilitates its nucleation at lower temperatures, aligning with the aging treatment objectives to maximize precipitation and strengthening [23]. Zhao et al. [24] reported that V (C, N) precipitation strengthening compensates for the reduction in strength due to the decreased pearlite content. In addition, nitrogen incorporation enhances the strength, plasticity, and toughness of steel. The controlled enhancement of nitrogen content facilitates the precipitation of V (C, N).

Conventional non-alloyed composites effectively mitigate the deterioration of thermal expansion properties degradation caused by excessive carbon, while simultaneously maximizing precipitation strengthening effects.

In this study, the research approach of “reducing carbon content and increasing nitrogen content to promote V (C, N) precipitation” was followed. To further maximize the precipitation effect, a cold deformation pretreatment was innovatively employed to introduce defects, which provided abundant nucleation sites for V (C, N) precipitation and effectively promoted the nucleation process [25,26]. Subsequently, the precipitation behavior of V (C, N) was regulated through various heat treatment processes. Meanwhile, compared with the limited contribution of a single strengthening mechanism for strength improvement, the synergistic effect of multiple strengthening mechanisms in this study significantly enhanced the comprehensive properties of the alloy. The impact of different strengthening mechanisms and the behavior of thermal expansion were investigated through a thorough microstructure evolution analysis. These findings achieve an effective improvement in the strength of the Fe-36Ni invar alloy while maintaining its excellent low thermal expansion properties and provide a theoretical foundation for the development of invar alloys with extraordinary comprehensive properties.

## 2. Material and Methods

### 2.1. Material Preparation and Heat Treatment Procedures

**Figure 1:**
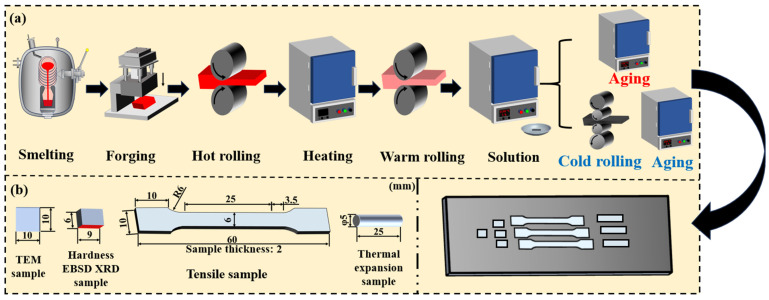
Schematic diagram of the (**a**) processes and (**b**) sampling.

Vacuum induction melting furnaces (KZG-5, Henan Kust Instrument Technology Co., Zhengzhou, Henan, China) were utilized to melt invar and V-N invar in argon and nitrogen atmospheres, respectively. Invar was a composition, while the V-N invar alloy contained vanadium and nitrogen as microalloying elements. Table 1 presents the chemical compositions of both invar alloys. The as-cast ingots obtained from smelting were held at 1150 °C for 1 h and then forged into flat ingots with a cross-sectional dimension of 26 mm × 40 mm. Afterwards, they were first hot rolled into plates with a thickness of 15 mm using a two-high reversing hot-rolling mill (Shanghai Electric SHMP Pulverizing & Special Equipment Co., Ltd., Shanghai, China) along the thickness direction at a rolling reduction of 40%, with start and finish temperatures of 1150 °C and 900 °C, respectively. The oxide layers formed on the surfaces were subsequently removed by electrical discharge machining (EDM, Beijing Andajianqi Digital Equipment Co., Ltd., Beijing, China). Then, the hot-rolled alloys were subjected to three passes of warm rolling at 650 °C, and the final thicknesses were reduced to 8 mm. The warm-rolled alloy plates were solution-treated at 1150 °C for 1 h, followed by water quenching. These solid solution samples were marked as “S”. Part of the S alloys were subjected to cold rolling with a 40% reduction rate and labeled as CR. Both S and CR samples were aged at 450 to 850 °C for 1–5 h. The aged alloys were named SA and CRA, respectively. The detailed processing routes are displayed in Figure 1a.

### 2.2. Material Characterization

As shown in Figure 1b, JEM-2000 transmission electron microscope (TEM, JEOL Ltd., Akishima, Tokyo, Japan), X-ray diffraction (XRD), electron backscattered diffraction (EBSD), and hardness, tensile, and thermal expansion samples were sectioned at one-third of the width from the edge of the alloy plates, with the sections parallel to the rolling direction. The observation surface for XRD, EBSD, and hardness tests corresponded to the longitudinal section along the plate thickness direction.

XRD measurements were conducted at room temperature using a DX2700B diffractometer (XRD, Dandong Haoyuan Instrument, Dandong, Liaoning, China) with a scanning rate of 2°/min over a 2θ range of 30–95°. The obtained diffraction patterns were analyzed using Jade (6.5) software to calculate distribution density based on the following equations [27,28]:

(1) $$\beta =4\varepsilon tan\theta +\frac{K\lambda }{dcos\theta }$$

(2) $$\rho =\frac{2\sqrt{3}\times \varepsilon }{b\times d}$$

where β represents the XRD peak broadening, λ is the wavelength of Cu K_α1_ radiation, K is a constant (0.9), b is the length of the Burgers vector (0.25787 nm), ε is the microstrain determined by the slope value of the linear fit plot of βcosθ and 4sinθ, and ρ is the dislocation density.

The EBSD samples were prepared by electrolytic polishing in a solution of 10% perchloric acid and 90% glacial acetic acid at 30 °C with a voltage of 25 V for 20 s. The EBSD measurement was carried out on the 7900F Field Emission Scanning Electron Microscope (SEM, JEOL Ltd., Akishima, Tokyo, Japan) with a step size of 0.15 μm. The Channel 5 (2019 v5.12) software was used to perform the data analysis. A TEM analysis was employed to observe microstructure and precipitates. Samples were initially ground manually to a thickness of approximately 50 µm, followed by punching 3 mm diameter thin films. Then they were electro-polished with 4% perchloric acid solution at −20 °C under a voltage of 75 V. The compositions of the precipitated particles were detected by energy-dispersive X-ray spectroscopy (EDS) attached to a JEM-2000 transmission electron microscope (TEM, JEOL Ltd., Tokyo, Japan).

For XRD, EBSD, and TEM analysis, each sample was subjected to repeated observations in multiple regions, and the results from multiple fields of view were statistically analyzed and averaged to ensure the reliability of microstructural parameters.

### 2.3. Material Properties

The hardness samples were tested with an HVHVS-1000A (Hengyi Instrument, Weifang, Shandong, China) Vickers hardness tester at a load of 100 g and a dwell time of 12 s. The hardness value for each sample was measured at least eight times and averaged. The dimensions of tensile samples are shown in Figure 1. The tensile tests were carried out via an INSTRON 5900 (Instron, Norwood, MA, USA) universal material testing machine, which was fitted with an extensometer that had a gauge length of 25 mm. The tests were performed at room temperature with a loading rate of 2 mm/min. To ensure measurement accuracy, at least three samples were tested for each condition. The typical tensile fracture surface morphology was examined via FE-SEM:7900F FESEM.

The thermal expansion measurements were carried out using a NETZSCH TMA402F3 (NETZSCH, Selb, Bavaria, Germany) thermal expansion tester in the temperature range of 20 to 300 °C at a heating rate of 5 °C/min. Three samples were tested for each condition. The CTE value was calculated using Formula (3):

(3) $$\alpha =\frac{∆L}{∆T}\frac{1}{L_{0}}$$

where α is the CTE, ∆L is the relative change in length, $L_{0}$ is the original length (25 mm), and ∆T is the temperature variation.

The results of multiple experiments on mechanical and physical properties are relatively consistent, demonstrating the stability of the experimental process and the reproducibility of the results.

## 3. Discussion

### 3.1. Mechanical Properties

**Figure 2:**
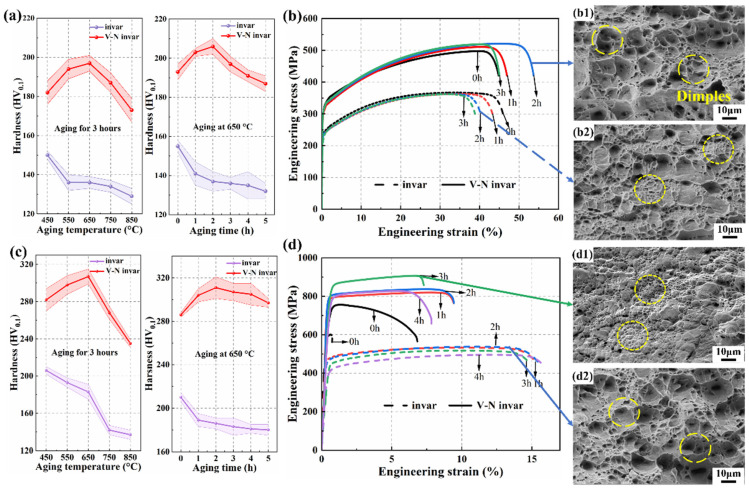
Variation in hardness, stress–strain curve, and fractograph of the invar and V-N invar at different processes. (**a**) Variation in hardness of SA samples; (**b**) the stress–strain curve and fracture morphology of the experimental alloy: (**b1**) V-N invar SA; (**b2**) invar SA; (**c**) Variation in hardness of CRA samples; (**d**) the stress–strain curve and fracture morphology of the experimental alloy: (**d1**) V-N invar CRA; (**d2**) invar CRA.

Figure 2 provides a visual comparison of the mechanical properties of invar and V-N invar alloys under different heat and deformation treatments. The temperature and time dependence of hardness is illustrated in Figure 2a. The results demonstrated that the V-N invar had a higher hardness than conventional invar, exhibiting an initial increase in hardness followed by a decrease as the aging temperature and time increased. The maximum hardness of 206 HV_0.1_ was achieved in the V-N invar alloy after aging at 650 °C for 2 h. However, the hardness of conventional invar gradually decreased during aging. This decline was primarily attributed to the absence of microalloying elements such as V and N. As the temperature increased or aging time extended, dislocations within the matrix underwent recovery and annihilation, accompanied by grain growth. These factors collectively resulted in the monotonically decreasing in hardness. In contrast, V-N microalloyed invar can form V (C, N) precipitation phases. During short-term aging, a significant amount of fine V (C, N) precipitated, and precipitation strengthening dominated, leading to an increase in hardness with a prolonged aging time. When aging time was excessively long, the precipitates coarsened, and the reduced dislocation density due to recovery caused the weakening effect to outweigh the precipitation strengthening, resulting in a decrease in hardness.

Figure 2b further demonstrates the mechanical properties of invar and V-N invar alloys aged at 650 °C. The V-N invar strength was also greater than that of invar, consistent with the hardness results and confirming the strengthening effect induced by microalloying. Specifically, invar exhibited a tensile strength (TS) of 361 MPa after aging at 650 °C for 2 h, whereas V-N invar attained a maximum TS of 520 MPa under the same aging process, while also exhibiting exceptional ductility. As shown in Figure 2(b1,b2), the fractographic analysis reveals typical ductile fracture features, including dimples and micropores. The size, depth, and uniformity of the dimple distribution are correlated with the material’s plasticity [29]. The tensile fracture morphology of V-N invar in the optimal aging regime exhibited larger and deeper dimples compared to invar. This observation aligns with the tensile results, which indicate that V-N invar demonstrates an exceptional combination of strength and plasticity.

The direct aging process had a limited impact on strength enhancement. Therefore, further investigation was carried out using a cold-rolling aging treatment. The temperature dependence of hardness for invar and V-N invar is shown on the left side of Figure 2c. For V-N invar, the hardness increased from 282 HV_0.1_ to 307 HV_0.1_ between 450 °C and 650 °C. However, a significant drop in hardness occurred at temperatures above 650 °C. The reason for the decreasing phenomenon is that the deformation energy stored during cold rolling drives the metastable structure into a steady state, while recrystallization causes the hardness to decrease dramatically [30]. The hardness of V-N invar aged at 650 °C increased with time, reaching a maximum of 311 HV_0.1_ after 2 h of aging, which is about 8.7% higher than the V-N invar CR sample.

The yield strength (YS) and TS of the V-N invar CR sample were 675 MPa and 757 MPa, respectively, representing an increase of 134% and 52.9% compared to the V-N invar S sample. This result demonstrates that cold rolling plays a critical role in significantly enhancing the strength of the invar alloy. Aging at 650 °C contributed to an increase in strength, with the V-N invar CRA sample aged for 3 h exhibiting the highest TS of 907 MPa. This value was about 19.8% higher than that of the V-N invar CR sample (757 MPa). The combined effects of V-N microalloying and cold-rolling aging yielded a more pronounced effect on strength enhancement. These findings were in accordance with the literature reports [11]. The fracture of the alloy following cold-rolling aging exhibited a characteristic ductile fracture. In the invar samples, larger and deeper dimples were associated with good plasticity, while the V-N invar samples exhibited smaller and shallower dimples, corresponding to poorer plasticity. These microstructural features are consistent with the tensile tests results.

### 3.2. Microstructure

#### 3.2.1. Microstructure Characterization

**Figure 3:**
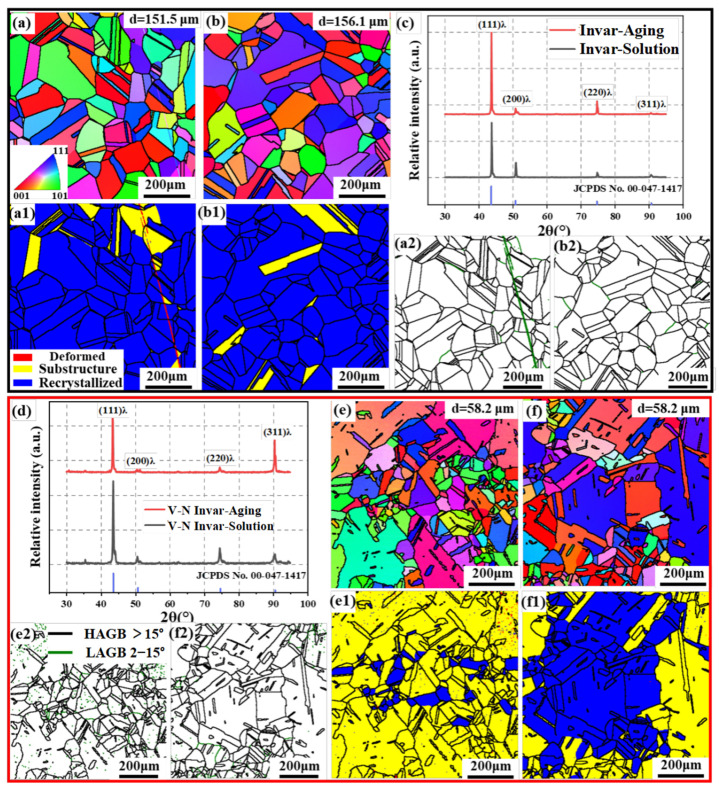
EBSD analysis results. (**a**) IPF map of invar S; (**a1**) GOS map of invar S; (**a2**) grain boundary map of invar S; (**b**) IPF diagram of invar SA; (**b1**) GOS map of invar SA; (**b2**) grain boundary map of invar SA; (**c**) XRD pattern of invar S and SA; (**d**) XRD pattern of V-N invar S; (**e**) IPF map of V-N invar S; (**e1**) GOS map of V-N invar S; (**e2**) grain boundary map of V-N invar S; (**f**) IPF diagram of V-N invar SA; (**f1**) GOS map of V-N invar SA; and (**f2**) grain boundary map of V-N invar SA.

The microstructures of solid solution, direct aging, cold rolling, and cold-rolling aging samples with optimal mechanical properties were analyzed to elucidate the mechanisms responsible for their improved mechanical properties. Figure 3 shows the EBSD results of S and SA samples in invar and V-N invar. As shown in Figure 3a,e, the grains of invar S and V-N invar S were equiaxed with grain sizes of 151.5 μm and 58.2 μm, respectively. V-N microalloying led to substantial grain refinement in the alloy. Due to the low stacking fault energy of austenite, a significant number of large-sized twins were formed in both invar and the V-N invar alloy matrix. For the invar SA and V-N invar SA sample, the grain size remained substantially constant compared to the S samples in both alloys, indicating that no grain growth occurred during the direct aging process. Therefore, the observed increase in strength after aging cannot be attributed to the grain size refinement. The analysis of the recrystallized tissue distribution indicates that the alloy samples in both the S and SA states are recrystallized, with almost no low-angle grain boundaries (LAGBs). Since LAGBs were formed through the slip and motion of dislocations, their fractions can qualitatively represent the distribution densities [31]. As illustrated in Figure 3(a2,b2,e2,f2), the LAGBs content in both the alloys after solid solution and direct aging were extremely low, indicating a minimal dislocation density at this stage.

The XRD patterns shown in Figure 3c,d indicate the presence of a single austenitic phase in all samples, with no detectable second phases. This is likely attributed to the small volume fraction of the second phases, which is not discernible against the diffraction peaks of austenite. Dislocations significantly influence the nucleation and growth of precipitates through their interactions, making the evaluation of dislocation density essential for understanding the strengthening mechanisms. Furthermore, the dislocation densities were quantitatively assessed using XRD data based on Equations (1) and (2). The relevant results are presented in Table 2.

During aging, thermally activated dislocation motions led to the rearrangement of dislocations during the aging process [32]. Consequently, in both invar SA and V-N invar SA samples, the dislocation densities were lower than those of the S samples. However, due to the pinning effects of grain boundaries and precipitates, V-N invar exhibited higher dislocation densities than invar alloys under all processing conditions. This finding aligned with the results of the grain boundary distribution, as the dislocation density of alloys after direct aging remained low. Based on the above analysis, the substantial increase in strength following the direct aging in V-N invar is closely associated with the precipitation strengthening that arises from the formation of precipitates.

**Figure 4:**
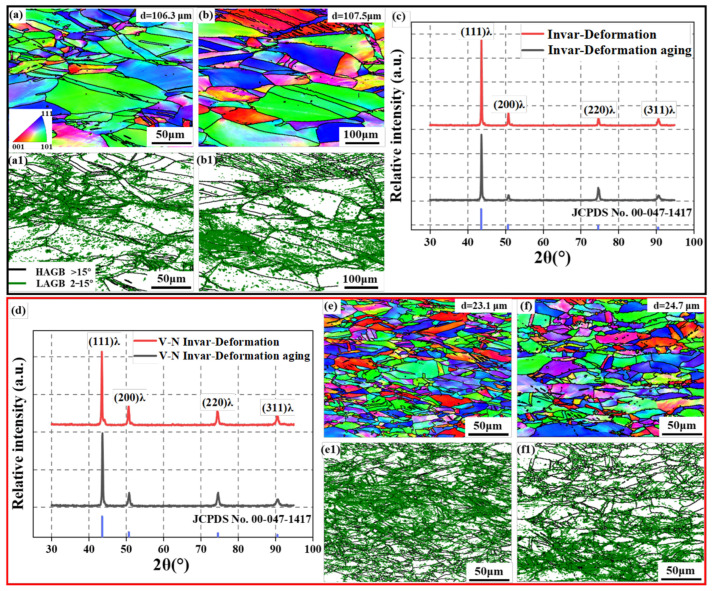
EBSD analysis results. (**a**) IPF map of invar CR; (**a1**) grain boundary map of invar CR; (**b**) IPF diagram of invar CRA; (**b1**) grain boundary map of invar CRA; (**c**) XRD pattern of invar S and SA; (**d**) XRD pattern of V-N invar S; (**e**) IPF map of V-N invar CR; (**e1**) grain boundary map of V-N invar CR; (**f**) IPF diagram of V-N invar CRA; and (**f1**) grain boundary map of V-N invar CRA.

Figure 4 shows the microstructures of samples subjected to cold rolling and the aging process. Cold rolling with a 40% reduction rate after the solid solution made the grain elongate along the deformation direction. After cold rolling, the grain sizes of invar and V-N invar were reduced to 106.3 μm and 23.1 μm, respectively. Compared with the S alloys, the grain size was obviously refined due to the fragmentation of large grains during the cold-rolling process. The refinement of the V-N invar was particularly noteworthy, achieving a 60.3% reduction in size compared to the solid-solution state, whereas invar experienced a reduction of only 29.8%. Additionally, cold rolling altered the grain texture from a random orientation in the solid-solution state to predominantly blue (<111>) and red (<001>) in the cold-rolling state. This texture evolution was consistent with the observations reported by Dong et al. [33] for invar alloys with varying cross-section reduction rations. As shown in Figure 4(a1,e1), the dislocations in the alloy after cold rolling were distributed around grain boundaries and also formed numerous aggregates within the grains. The high density of dislocations significantly enhanced the strength of the V-N invar. However, the accumulation of residual stresses resulting from the large number of dislocations reduced the alloy’s plasticity, leading to a marked decrease in elongation. This aligns with the stress–strain findings. During the cold-rolling aging process, a significant number of dislocations and grain boundaries acted as nucleation sites for precipitates, facilitating their nucleation and growth. These precipitates, in turn, effectively pinned the dislocations and stabilized grain boundaries. Consequently, the grain size of the CRA samples (24.7 μm) increased slightly compared with the CR samples (23.1 μm), while the dislocation density remained at a high level. XRD patterns in Figure 4c,d confirm that both alloys exhibit a single-phase austenitic structure, and no phase transformation or precipitate diffraction peaks were observed after the cold-rolling process. The quantitative results of the dislocation density for the two groups of alloys, following cold rolling and aging, are presented in Table 3. These values are consistent with the distribution of LAGBs. The combination of significant grain refinement and an increased dislocation density is identified as the dominant mechanism for the enhanced mechanical strength of the alloys.

#### 3.2.2. Precipitation Phases Characterization

**Figure 5:**
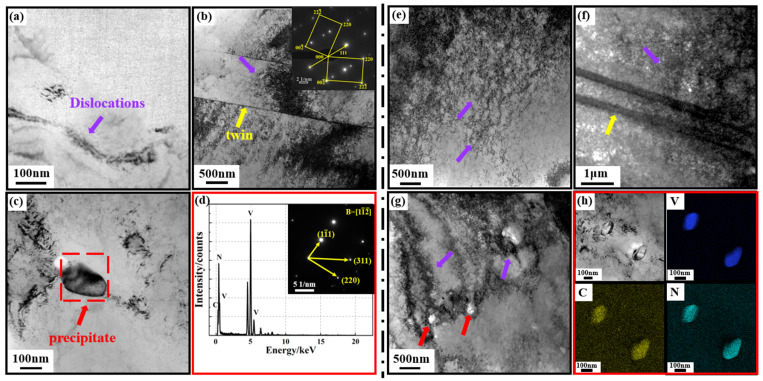
TEM morphologies of V-N invar. (**a**,**b**) Morphology diagram of twin and dislocation on SA sample; (**c**) morphology diagram of precipitated phase on SA sample; and (**d**) corresponding diffraction pattern and EDS of the phase marked with a red box in (**c**). (**e**–**g**) Morphology diagram of twin, dislocation, and precipitated phase on CRA sample; (**h**) precipitated phase and corresponding EDS mapping.

Combined with the previous analysis, the significant increase in V-N invar strength was closely related to synergistic effects of fine grain, dislocation, and precipitation strengthening. Therefore, further exploration of the interactions among alloy precipitation phases, dislocations, and grain boundaries is essential. Figure 5 illustrates the TEM images of dislocations, twins, and precipitates in V-N invar SA and V-N invar CRA samples. As shown in Figure 5a, dislocations in the SA samples were sparsely distributed, consistent with the EBSD results. Figure 5b illustrates the large, annealed twin in the V-N invar SA sample, and this was confirmed as a twin based on diffraction spot calibration. Both twins and dislocations were classified as matrix defects, and both significantly influenced the mechanical properties of alloys [34]. The dislocation motion was hindered by twin crystals, resulting in dislocation aggregations near these crystals. Additionally, the twins and the atoms arranged in various orientations adjacent to twins disrupted the original slip system of dislocations, forcing dislocations to continuously change their slip directions to facilitate motion [25]. Ultimately, in the V-N invar SA sample with a low dislocation density, the presence of twins resulted in localized, uneven dislocation distributions and the formation of a tangled structure. Additionally, precipitated phases effectively hindered the dislocation motion by acting as pinning sites. Figure 5c illustrates that the precipitated phase in the V-N invar SA sample was identified as V (C, N), based on the EDS analysis and diffraction pattern calibration in Figure 5d.

The CRA sample exhibited a significantly higher dislocation density and a more pronounced dislocation entanglement structure compared to the SA sample, as shown in Figure 5e,f. Additionally, the number of precipitated phase particles also rose considerably (Figure 5g), and the EDS presented in Figure 5h indicated that precipitates were V (C, N). Almost all of the precipitated phase particles were observed near the dislocations in both direct and cold-rolling aging samples, indicating that V (C, N) preferentially nucleated at the dislocation sites. The rearrangement and annihilation of dislocations during the solution and aging resulted in a reduced density of dislocations, and nucleation sites for V (C, N) were subsequently decreased. Conversely, the high dislocation density introduced by cold-rolling aging provided a driving force for V (C, N) nucleation and growth. As a result, the CRA sample contained a greater number of V (C, N) particles with a smaller size. These precipitates effectively pinned the dislocations and impeded their movement, resulting in the formation of the dislocation tangle structure. The larger volume fraction of precipitates and higher dislocation density are the primary factors contributing to significant increase in the strength of V-N invar. At the same time, inevitably, the interaction between dislocations and precipitation phases, as well as among dislocations, hinder dislocation movement, thereby reducing plasticity. This phenomenon accounts for the lower plasticity observed in the V-N invar and ordinary invar CRA samples compared to their SA samples.

#### 3.2.3. Microstructure Evolution

**Figure 6:**
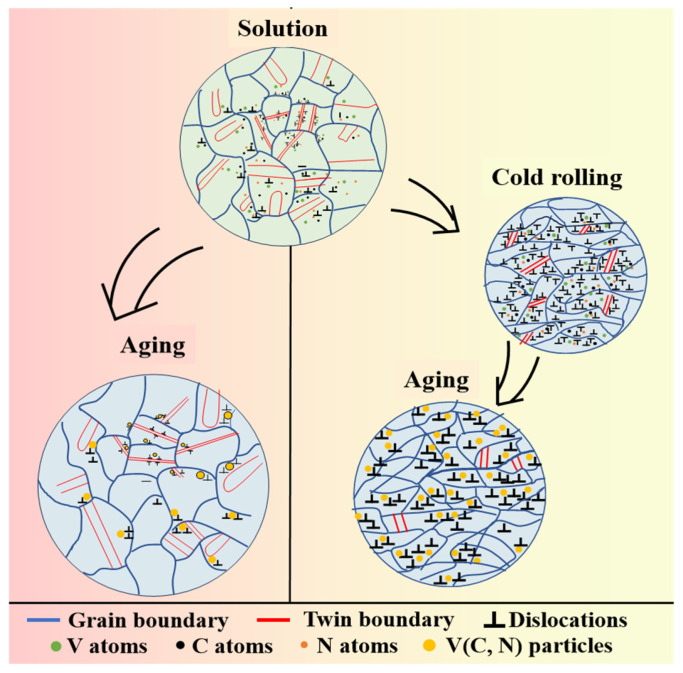
Schematic of microstructure evolution of V-N invar treated by different processes.

The microstructure evolution of V-N invar SA and CRA samples is presented in Figure 6. The V-N invar S sample exhibited larger grain sizes and a greater number of large twins within the matrix but a reduced number of dislocations. The solution treatment at 1150 °C enabled the dissolution of all large-sized precipitates generated during hot rolling into the matrix, promoting the subsequent formation of small-sized precipitates during aging. In the direct aging process, the dislocation density was further reduced due to thermal motion, and this resulted in fewer V (C, N) nucleation sites and precipitated phase. The V-N invar CR samples, cold rolled at a 40% deformation rate, exhibited significant grain refinement and an increased dislocation density. This elevated dislocation density reduced the free energy required for the second-phase nucleation, thereby accelerating the nucleation rate of fine V (C, N) grains within the matrix of the V-N invar CRA samples. These fine V (C, N) particles, along with twins and grain boundaries, impeded the dislocation movement. In contrast, Yang et al. [27] demonstrated that nanoparticles significantly hinder dislocation motion while simultaneously promoting dislocation proliferation. As a result, the dislocation density in the V-N invar CRA samples remained comparable to that in CR samples. The pinning effect of V (C, N) particles on the grain boundaries inhibited grain coarsening, resulting in a minimal change in grain size before and after aging in V-N invar.

### 3.3. Evaluation of Strengthening Mechanism

In order to quantitatively assess the contribution of various strengthening mechanisms to the YS of alloys, a structure-based strengthening calculation model was employed to estimate the strength of V-N invar SA and CRA samples, as described by Equation (4) [19]. SA-2 h (650 °C/2 h) and CRA-3 h (650 °C/3 h) represented the optimal performance states under each respective process. By using the same calculation methods, we can effectively compare the differences in strengthening mechanisms between direct aging and cold-rolling aging:

(4) $$\sigma_{0.2}=\sigma_{0}+\sigma_{ss}+\sigma_{g}+{\sigma_{dis}+\sigma }_{p}$$

where $\sigma_{0}$ is the matrix lattice resistance and the Peierls–Nabarro stress (54 MPa), and $\sigma_{ss}$, $\sigma_{g}$, $\sigma_{dis}$, and $\sigma_{p}$ are the contributions from solution strengthening, grain refinement strengthening, dislocation strengthening, and precipitation strengthening, respectively.

The strengthening from elements and precipitates will be individually discussed in the following sections. The σ_ss_ can be evaluated by Equations (5)–(9) [35,36]:

(5) $$\log\frac{\omega_{\left[V\right]}\omega_{\left[C\right]}}{x}=6.72-\frac{9500}{T}$$

(6) $$\log\frac{\omega_{\left[V\right]}\omega_{\left[N\right]}}{1-x}=3.36-\frac{8700}{T}$$

(7) $$\frac{\omega_{V}-\omega_{\left[V\right]}}{\omega_{C}-\omega_{\left[C\right]}}=\frac{50.9414}{12.011x}$$

(8) $$\frac{\omega_{V}-\omega_{\left[V\right]}}{\omega_{N}-\omega_{\left[N\right]}}=\frac{50.9414}{14.0067\left(1-x\right)}$$

(9) $$\sigma_{ss}=4570\omega_{{\left[C\right]}^{*}}+4570\omega_{{\left[N\right]}^{*}}+3\omega_{{\left[V\right]}^{*}}+32\omega_{\left[Mn\right]}$$

where $\omega_{M}$ (M = C, N, V, and Mn) is the mass fraction of element M, $\omega_{\left[M\right]}$ ([M] = [C], [N], [V], and [Mn]) corresponds to the mass fraction of element [M] dissolved in the matrix, x represents the effective activity of VC in the VC_x_N_(1−x)_ phase, T is the solution temperature, K; and $\omega_{\left[M\right]}$ ([M*] = [C*], [N*], and [V*]) denotes the difference between $\omega_{\left[M\right]}$ and V (C, N) precipitation content. Furthermore, the element Mn is an austenite-forming element. According to Formulas (5)–(9) and the EDS of the precipitation phase, the increase in the strength of SA and CRA samples by solid solution strengthening are 50.7 MPa and 37.4 MPa, respectively.

$\sigma_{g}$ represents the amount of the grain boundary strengthening, which is related to the size of the grains. The value can be obtained via the Hall–Petch formula [37]:

(10) $$\sigma_{g}=k_{y}d^{-\frac{1}{2}}$$

where $k_{y}$ represents the strengthening coefficient (24.7 MPa mm^0.5^). The grain sizes of SA and CRA samples are 58.5 μm and 23.1 μm (Figure 3 and Figure 4), respectively. Therefore, the corresponding values of $\sigma_{g}$ are 102.1 MPa and 162.5 MPa.

Dislocation strengthening can be described using the Bailey–Hirsch equation [38]:

(11) $$\sigma_{dis}=M\alpha Gb\rho^{\frac{1}{2}}$$

where M represents the Taylor factor (3.06), α is a constant depending on crystal structure and 0.5 is used in this work, b is the Burgers vector (0.25787 nm), G represents the shear modulus of the matrix (80 GPa), and *ρ* is the dislocation density (Table 2 and Table 3, the data from the repeated experiments can be found in Tables S1, S3, and S5 of the Supplementary Materials.). Thus, the $\sigma_{dis}$ can be evaluated as 107.9 MPa and 311.8 MPa for SA and CRA samples, respectively.

The formula for calculating precipitation strengthening is shown below [19]:

(12) $$\sigma_{p}=\left(\frac{k}{d}\right)\times f_{v}^{\frac{1}{2}}\times \ln\left(\frac{d}{b}\right)$$

where k is a constant with a value of 5.9 N/m, $f_{v}$ is the volume fraction of the precipitated phase, and d is the average size of the precipitated phase. Based on measurement and statistic calculations, the f and d for the V (C, N) particles were found to be 0.025%, 312.2 nm and 0.406%, and 60.8 nm, respectively. These give rise to a $\sigma_{p}$ of 6.1 MPa and 270.0 MPa for SA and CRA samples, respectively.

**Figure 7:**
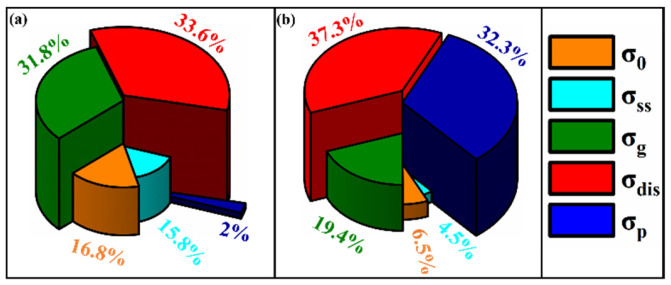
Strengthening contribution of various mechanisms of SA-2 h and CRA-3 h samples on V-N invar. Strengthening contribution of various mechanisms of V-N invar at different processes. (**a**) SA-2 h; (**b**) CRA-3 h.

Table 4 presents $\sigma_{0.2}$ and various strengthening mechanisms of the V-N microalloyed invar alloys under different processing treatments. The data from the repeated experiments can be found in Tables S2, S4, and S6 of the Supplementary Materials. The pie charts in Figure 7 further illustrate the percentage contributions of these mechanisms. The results indicate that the theoretically calculated values for the SA and CRA samples generally align with the experimental values, highlighting the primary strengthening mechanisms responsible for the substantial increase in the strength of V-N invar alloys. For the SA samples, the main strengthening mechanisms are dislocation and fine grain strengthening, while for the CRA samples, dislocation strengthening and precipitation strengthening are the dominant factors. The contribution of precipitation strengthening in the CRA samples increases significantly, indicating that aging after cold rolling enhances the precipitation strengthening effect of V (C, N).

### 3.4. Thermal Expansion Behavior

**Figure 8:**
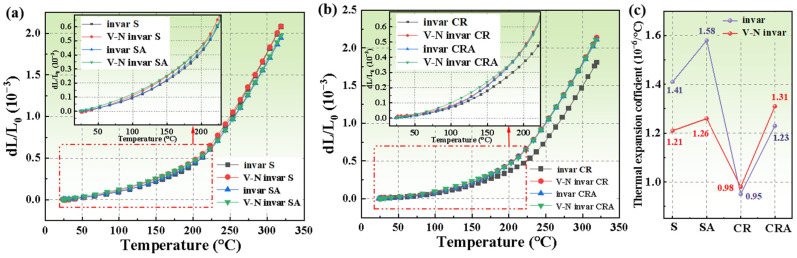
(**a**) Thermal expansion curves of samples under direct aging; (**b**) thermal expansion curves of samples under cold-rolling aging; and (**c**) CTE values of samples in the range of 20~100 °C treated by different processes.

Figure 8 presents the thermal expansion curves for invar and V-N invar alloys under various processing routes and the CTE values calculated according to Equation (3) in the temperature range of 20 to 100 °C. As shown in Figure 8a, the thermal expansion curves of the alloys exhibited a trend of gradual growth below the Curie temperature, followed by a steep increase in slope above the Curie temperature. This behavior originates from the antiferromagnetic ordering below the Curie temperature, which induces a lattice contraction that counteracts thermal expansion, thereby resulting in a reduced CTE value [39]. Overall, the thermal expansion curves of invar were lower than those of V-N invar alloys for both the solid solution and direct aging processes, as the addition of alloying elements such as C, V, and N inevitably increased the CTE value [40]. Figure 8b illustrates the thermal expansion curves of invar and V-N invar alloys subjected to cold rolling and the aging process. These curves also demonstrate a similar trend of a slower slope at low temperatures and a sharply steeper slope at high temperatures. The low-temperature thermal expansion curves of alloys after cold rolling and the aging process were nearly identical. However, as the temperature increased, the change in the length of V-N invar alloys increased significantly, leading to a deterioration in thermal expansion properties.

As illustrated in Figure 8c, the CTE of the alloys exhibited an increasing trend after aging, with the highest value observed in the direct aging condition and the lowest in the cold-rolling condition. According to the literature [41], the presence of defects such as dislocations disrupted the short-range order of atoms, thereby reducing the CTE of alloys. Cold-rolled invar and V-N invar alloys exhibited the highest dislocation density of 1.69 × 10^13^ and 10.74 × 10^13^, resulting in a low CTE of 0.95 × 10^−6^/°C and 0.98 × 10^−6^/°C, respectively.

Thermal expansion properties are influenced by phase compositions, crystal defects, and the presence of precipitated particles [42,43]. Therefore, the incorporation of alloying elements into V-N invar alloys significantly contributes to higher CTE compared to invar alloys. Considering the V-N invar alloy as a binary system consisting of a matrix and a precipitated phase, the CTE can be expressed using the following equation [34]:

(13) $$\alpha =\alpha_{A}\cdot \left(1-f_{p}\right)+\alpha_{p}\cdot f_{p}$$

where $\alpha_{A}$ and $\alpha_{p}$ are the CTE value of the austenite and the V (C, N) particles, respectively. $f_{p}$ is the volume fraction of the V (C, N) particles.

With aging, V (C, N) particles gradually precipitate from supersaturated austenite, decreasing the concentration of alloy elements (such as C, V, and N) in the matrix, which subsequently diminishes the CTE value of austenite. Concurrently, due to the high CTE of the carbonitrides, the CTE value associated with the precipitated V (C, N) particles significantly increased. Accordingly, the CTE value of the V-N invar CRA sample, containing a high-volume fraction of V (C, N) particles, is theoretically expected to be significantly greater than that of the SA sample. However, the experimental results were markedly inconsistent with the theoretical predictions. These discrepancies can be attributed to the increase in dislocation density (CRA: 9.76 × 10^13^ SA: 1.17 × 10^13^), which is significantly enhanced by the cold-rolling process. This, in turn, affects the spontaneous magnetization and hysteresis expansion coefficient of the invar alloys, ultimately leading to a reduction in CTE [44]. The combined effect of these factors results in a low CTE observed in the CR V-N invar alloys.

**Figure 9:**
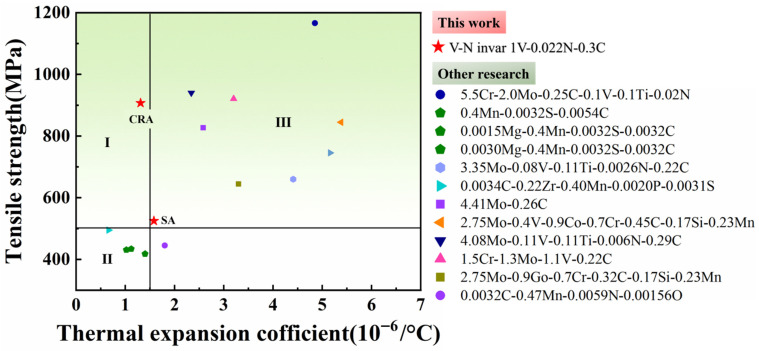
Comparison of the thermal expansion coefficient and tensile strength of the V-N invar alloy in this study with those of other related invar alloys from previous research.

To further elucidate the contributions of our study, the tensile strength and CTE of V-N invar alloys are compared to the published results in Figure 9.

Using the comprehensive performance cutoffs of a 500 MPa strength and 1.5 for the coefficient of thermal expansion, the invar alloys in the literature are broadly categorized into several zones. Zone I represents the region of high-strength and low-thermal expansion invar alloys that are currently desired; Zone II represents invar alloys prepared using emerging additive manufacturing technologies, which usually have a low-strength and very low coefficient of thermal expansion; and Zone III represents a class of invar alloys that achieve high strength at the expense of thermal expansion through the addition of a high content of alloying elements. The V-N invar alloys prepared in this paper, after cold rolling and aging, demonstrate an excellent combination of high-strength and low-thermal expansion properties.

## 4. Conclusions

Based on the evolution of mechanical properties, microstructures, and thermal expansion properties in invar and V-N microalloyed invar during direct aging and cold-rolling aging, the following conclusions are drawn:

(1) The V-N microalloyed invar alloys exhibited superior mechanical properties compared to invar alloys, under both direct aging and post-deformation aging treatments. The V-N microalloyed invar alloys achieved a tensile strength of 907 MPa after cold-rolling aging at 650 °C for 3 h.
(2) During direct aging, coarse V (C, N) particles contributed to limited precipitation strengthening, resulting in a relatively moderate strength enhancement in V-N microalloyed invar alloys. In contrast, cold-rolling aging significantly generated a dislocation density that promoted the intensive nucleation of V (C, N) fine precipitates. The synergistic combination of dislocation strengthening and precipitation strengthening mechanisms enabled substantial strength improvement in cold-deformed V-N microalloyed invar alloys.
(3) V-N microalloyed invar alloys exhibited an increased CTE value due to finer grains and the precipitation of V (C, N) to invar alloys. However, the high density of dislocations induced by cold rolling resulted in a low CTE of 1.31 × 10^−6^/°C.

## Figures and Tables

**Table 1:** Chemical compositions of invar alloys (wt. %).

Scheme	C	Si	Mn	S	P	V	N	Ni	Fe
invar	0.022	0.17	0.31	0.001	0.006	-	-	36.10	Bal
V-N invar	0.300	0.16	0.31	0.001	0.008	1.0	0.022	36.02	Bal

**Table 2:** Dislocation density and ε of S and SA samples of invar and V-N invar.

	Invar S	V-N Invar S	Invar SA	V-N Invar SA
*ε* (microstrain)	0.0195	0.1000	0.0025	0.0510
Dislocation density (m^−2^)	1.70 × 10^12^	2.30 × 10^13^	2.18 × 10^11^	1.17 × 10^13^

**Table 3:** Dislocation density and ε of CR and CRA samples of invar and V-N invar.

	Invar CR	Invar CRA	V-N Invar CR	V-N Invar CRA
*ε* (microstrain)	0.1345	0.1015	0.1824	0.1759
Dislocation density (m^−2^)	1.69 × 10^13^	1.28 × 10^13^	10.74 × 10^13^	9.76 × 10^13^

**Table 4:** Strengthening contribution of various mechanisms of V-N SA-2 h and CRA-3 h alloys.

Samples	σ_0/_MPa	σ_ss_/MPa	σ_g_/MPa	σ_dis_/MPa	σ_p_/MPa	σ_cal_/MPa	σ_exp_/MPa
SA-2 h	54	50.7	102.1	107.9	6.1	320.8	315
CRA-3 h	54	37.4	162.5	311.8	270.0	835.7	835

## Data Availability

The original contributions presented in this study are included in the article. Further inquiries can be directed to the corresponding authors.

## Supplementary Materials

The following supporting information can be downloaded at: https://www.mdpi.com/article/10.3390/ma18173934/s1, Table S1: Dislocation density and ε of SA and CRA samples of invar and V-N invar; Table S2: Strengthening contribution of various mechanisms of V-N SA-2h and CRA-3h alloys; Table S3: Dislocation density and ε of SA and CRA samples of invar and V-N invar; Table S4: Strengthening contribution of various mechanisms of V-N SA-2h and CRA-3h alloys; Table S5: Dislocation density and ε (mean ± error) of SA and CRA samples of invar and V-N invar; Table S6: Strengthening contribution (mean ± error) of various mechanisms of V-N SA-2h and CRA-3h alloys.
