# Supplementary material for: Microstructure Evolution and Strengthening Mechanism of V-N Microalloyed Invar Alloy Processed by Mechanical Heat Treatment
Source: Materials (Basel). 2025 Aug 22;18(17):3934. doi: 10.3390/ma18173934 (PMC12429711; doi:10.3390/ma18173934)
Supplement: Supplementary file 1 [file materials-18-03934-s001.zip › materials-3784070-supplementary.pdf]

sample 1

Table S1. Dislocation density and ε of SA and CRA samples of invar and V-N invar.

	invar SA	V-N invar SA	invar CRA	V-N invar CRA
ε (micro strain)	0.0023	0.0475	0.0979	0.1712
Dislocation density(m^{-2})	1.99×10^{11}	1.10×10^{13}	1.22×10^{13}	9.31×10^{13}

Table S2. Strengthening contribution of various mechanisms of V-N SA-2h and CRA-3h alloys.

Samples	σ_0/MPa	σ_{ss}/MPa	σ_g/MPa	σ_{dis}/MPa	σ_p/MPa	σ_{cal}/MPa	σ_{exp}/MPa
SA-2h	54	48.7	100.8	104.8	6.2	314.5	312
CRA-3h	54	36.9	164.7	304.6	267.9	828.0	826

sample 2

Table S3. Dislocation density and ε of SA and CRA samples of invar and V-N invar.

	invar SA	V-N invar SA	invar CRA	V-N invar CRA
ε (micro strain)	0.0027	0.0539	0.1133	0.1813
Dislocation density(m^{-2})	2.36×10^{11}	1.25×10^{13}	1.42×10^{13}	9.86×10^{13}

Table S4. Strengthening contribution of various mechanisms of V-N SA-2h and CRA-3h alloys.

Samples	σ_0/MPa	σ_{ss}/MPa	σ_g/MPa	σ_{dis}/MPa	σ_p/MPa	σ_{cal}/MPa	σ_{exp}/MPa
SA-2h	54	52.8	101.4	109.7	6.8	324.7	320
CRA-3h	54	39.1	160.4	313.4	280.4	847.4	843

Table S5. Dislocation density and ε (mean±error) of SA and CRA samples of invar and V-N invar.

	invar SA	V-N invar SA	invar CRA	V-N invar CRA
ε (micro strain)	0.0025±0.0002	0.0508±0.0027	0.1042±0.0066	0.1761±0.0041
Dislocation density($\times10^{13}m^{-2}$)	0.02174±0.0015	1.1753±0.0614	1.3063±0.0810	9.6437±0.2387

Table S6. Strengthening contribution (mean±error) of various mechanisms of V-N SA-2h and CRA-3h alloys.

Samples	σ_0/MPa	σ_{ss}/MPa	σ_g/MPa	σ_{dis}/MPa	σ_p/MPa	σ_{cal}/MPa	σ_{exp}/MPa
SA-2h	54±0	50.7±1.7	101.5±0.5	107.5±2.0	6.4±0.3	320.0±4.2	316±3.3
CRA-3h	54±0	37.8±0.9	162.54±1.7	309.9±3.9	272.8±5.5	837.0±8.0	835±6.9
